# Case Report: Neuropsychiatric and cognitive phenomena in a highly complex patient affected by psychiatric and oncological diseases

**DOI:** 10.3389/fpsyt.2026.1904104

**Published:** 2026-09-09

**Authors:** Anna Borghesani, Katiuscia Trottner, Fabian Max Wedmann, Bryan Bertoldi, Luca Tondulli, Mohsen Farsad, Alessandro Spimpolo, Filippo Boschello, Andreas Conca

**Affiliations:** 1 Department of Psychiatry, Bolzano Hospital, Bolzano, Italy; 2 University Hospital of Psychiatry, Psychotherapy and Psychosomatics, Paracelsus Medical University, Salzburg, Austria; 3 Psychological Service, Bolzano Hospital, Bolzano, Italy; 4 Department of Oncology, Bolzano Hospital, Bolzano, Italy; 5 Department of Nuclear Medicine, Bolzano Hospital, Bolzano, Italy

**Keywords:** akathisia, cancer, case report, cognitive dysfunction, depression, encephalopathy, immune checkpoint inhibitors, lenvatinib

## Abstract

**Background:**

The introduction of innovative oncological therapies, such as immune checkpoint inhibitors (ICIs) and multikinase inhibitors (TKIs), has improved the prognosis of many malignancies but has introduced complex neuropsychiatric adverse events. This case report describes the management of a complex psycho-organic syndrome occurring in a patient treated with the combination of pembrolizumab and lenvatinib.

**Case presentation:**

A 69-year-old woman, with a history of anxious-depressive disorder and recurrent endometrial carcinoma, was admitted for the onset of a sub-confusional state, severe psychomotor retardation, akathisia, and tremors. These symptoms appeared in temporal relationship with the initiation of combined pembrolizumab-lenvatinib therapy. Diagnostic investigations (CT, MRI, FDG-PET, and CSF analysis) excluded brain metastases or primary neuroinflammatory processes, revealing diffuse cortical hypometabolism consistent with metabolic-iatrogenic encephalopathy.

**Management:**

The therapeutic strategy involved a multidisciplinary approach based on the precautionary suspension of oncological drugs and a structured pharmacological washout. Flumazenil was administered with diagnostic-therapeutic success to correct GABAergic redundancy, and pramipexole was introduced to treat extrapyramidal manifestations and akathisia.

**Conclusions:**

This case highlights how overlapping immunological, vascular, and neuropharmacological effects can generate reversible encephalopathic patterns in complex oncological patients. Symptom resolution through psychopharmacological modulation and suspension of targeted treatment alone underscores the critical importance of active neuropsychiatric surveillance and close interdisciplinary collaboration between oncologists and psychopharmacologists.

## Introduction

This case report analyzes the clinical course of a female patient affected by an abdominal recurrence of high-grade serous endometrial carcinoma, BRCA wild-type, receiving second-line treatment with an anti-PD1 monoclonal antibody and a tyrosine kinase inhibitor (TKI). The patient had also been followed for approximately three years by local psychiatric services for an anxious-depressive syndrome.

We believe this case is of particular interest due to the intersection between innovative oncological treatment and the onset of a polymorphous and complex psycho-organic presentation, requiring a highly individualized clinical approach and the coordinated involvement of various specialists.

While the co-existence of oncological malignancies and psychiatric disorders is highly frequent in clinical practice, systematic data regarding the safety, tolerability, and potential drug-drug interactions when psychotropic agents are concomitantly used with these specific novel oncological regimens (i.e., combined immune checkpoint inhibitors and tyrosine kinase inhibitors) remain limited.

To contextualize the case within available evidence, a comprehensive narrative literature review was conducted to identify relevant clinical cases and observational studies. The search was performed across major biomedical databases (PubMed/MEDLINE, Scopus, Web of Science) and Google Scholar to find any non-indexed contributions. Five relevant articles were identified, all consisting of case reports, describing neurological or psychiatric manifestations associated with pembrolizumab (n=5) or lenvatinib (n=2) treatment. The aim of this work is to discuss the potential association between this oncological treatment and the emergence of a psycho-organic syndrome, analyzing the patient’s clinical course and literature data, and to emphasize the importance of an interdisciplinary approach in managing neuropsychiatric adverse events in oncology.

## Case presentation

In July 2024, a 69-year-old woman was admitted to the sub-acute section of the Psychiatric Service for Diagnosis and Care (*Servizio Psichiatrico di Diagnosi e Cura* - SPDC) with a three-year history of anxious-depressive disorder and abdominal recurrence of BRCA-positive endometrial carcinoma. Her medical history included osteoporosis with prior fractures, osteoarthritis, cholecystectomy for prior cholelithiasis, and hiatal hernia. The patient was described as a previously active person, with a good social network and independence in daily activities.

### Pre-admission clinical history

Since 2021, the patient had received various psychopharmacological treatments for anxious-depressive symptoms, including antidepressants (citalopram 20 mg/day, vortioxetine 10 mg/day, escitalopram 15 mg/day), anxiolytics (Delorazepam 4 mg/day and pregabalin 75 mg/day), and low-dose atypical antipsychotics used as augmentation strategies (olanzapine 2,5 mg/day, brexpiprazole 2 mg/day). Clinical response remained incomplete, with fluctuating affective symptoms and persistent residual anxiety and emotional vulnerability.

In April 2023, following extensive peritoneal progression after standard first-line chemotherapy, the patient initiated combination therapy with pembrolizumab and lenvatinib. This regimen yielded favorable radiological, biochemical, and clinical responses, subsequently enabling debulking surgery and hyperthermic intraperitoneal chemotherapy (HIPEC) with mitomycin in September 2023. Combined therapy was resumed in October 2023.

Over time, her anxious-depressive symptoms were strongly associated with her oncological status, with exacerbations during diagnostic-therapeutic phases. With the introduction of biological therapy, her condition progressively worsened, manifesting as akathisia and a sub-confusional state, requiring hospitalization for diagnostic investigation and therapeutic optimization.

### Status at admission

Upon admission, a targeted physical and bedside neurological evaluation was performed. The patient was conscious but disoriented in time and space, fluctuating between a sub-confusional state and severe psychomotor retardation. Speech was severely slowed, dysarthric, and hypophonic, though comprehension remained grossly preserved. Cranial nerve assessment was unremarkable, and no gross motor deficits, sensory loss, or hemispheric focal signs were detected. However, a prominent, generalized fine peripheral tremor was observed at rest and during posture, accompanied by severe akathisia that prevented the patient from maintaining any stable position. Deep tendon reflexes were symmetric, and plantar responses were flexor bilaterally. Meningeal signs were absent. Vital signs showed mild hemodynamic instability with a tendency toward hypotension (blood pressure 105/65 mmHg) and a regular heart rate of 72 bpm. No psychotic ideation or suicidal intent was observed; sleep was reported as preserved. Her home medication regimen included delorazepam 4 mg/day, pregabalin 75 mg ter in die (TID), escitalopram 15 mg/day, olmesartan 40 mg/day, prednisone 5 mg/day, oxycodone+paracetamol 325 + 5 mg/day, lenvatinib 10 mg/day (reduced dose for non-optimal performance status), and pembrolizumab 200 mg IV every 21 days.

### Clinical course and management

#### First phase: diagnostic clarification and guided washout

During the first week, the patient exhibited severe psychomotor retardation, marked tendency to remain in bed (clinophilia), reduced speech, and attentional difficulties. An urgent brain CT was negative for acute lesions. Mild hypofolatemia was corrected with folic acid 5 mg/day, and omega-3 (1 g TID) was introduced for neurotrophic support. Given the recent history of clinical deterioration following multiple psychotropic and oncological therapies, a structured pharmacological washout was initiated to facilitate diagnostic clarification under the suspicion of multifactorial iatrogenic encephalopathy (resulting from sedative interactions, immunotherapy, and somatic vulnerability). Benzodiazepines and pregabalin were cross-tapered to diazepam to allow controlled withdrawal and limit rebound symptoms. Escitalopram was discontinued, and intravenous flumazenil was subsequently administered in fractionated doses to assess the contribution of residual GABAergic effects and facilitate clinical reassessment. While serotonergic exposure was discontinued, adaptive serotonergic changes may have persisted beyond drug withdrawal. The positive response, with improved consciousness and orientation, strongly supported an iatrogenic benzodiazepine contribution. After a multidisciplinary oncological evaluation, lenvatinib was suspended and the next pembrolizumab dose postponed.

#### Second phase: neurodiagnostic evaluation and dopaminergic support

In the second week, consciousness and responsiveness improved further, supported by additional flumazenil administration. Following the completion of benzodiazepine withdrawal, akathisia, tremors, and extrapyramidal features persisted. Consequently, dopaminergic treatment with pramipexole was initiated at 0.18 mg/day and subsequently titrated to 0.18 mg TID. Its administration aimed to support dopaminergic neurotransmission to facilitate the recovery of motor initiative, motivation, and psychomotor fluidity. The drug was also employed *ex adiuvantibus* to confirm the role of a functional dopaminergic deficit as a contributing factor to the clinical picture. This approach proved clinically effective, resulting in progressive reactivation.

**Figure 1:**
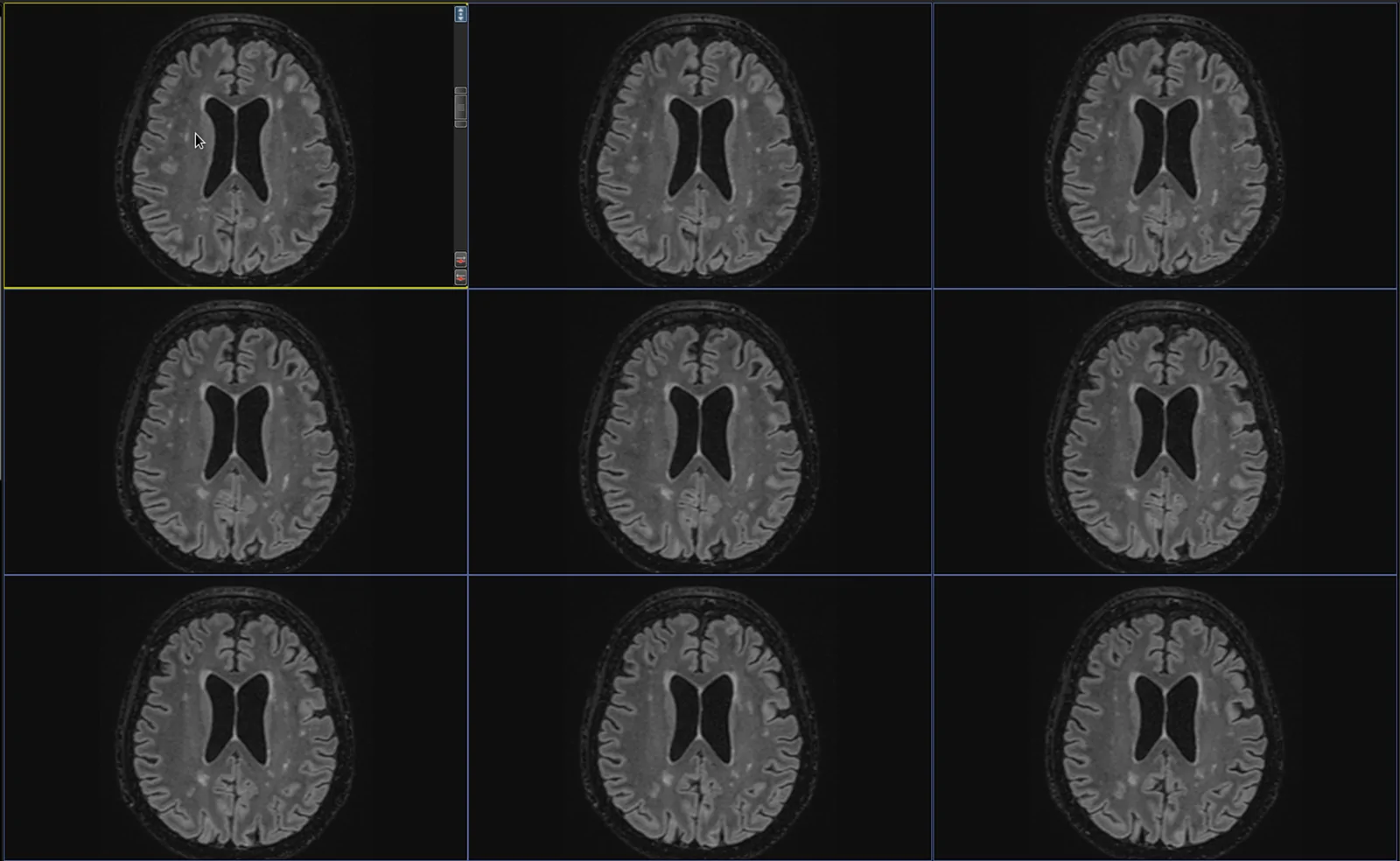
Brain magnetic resonance imaging (MRI). Axial T2-weighted/fluid-attenuated inversion recovery (FLAIR) sequences demonstrating multiple, partially confluent areas of hyperintensity in the subcortical, deep, and periventricular white matter, consistent with non-specific gliosis.

**Figure 2:**
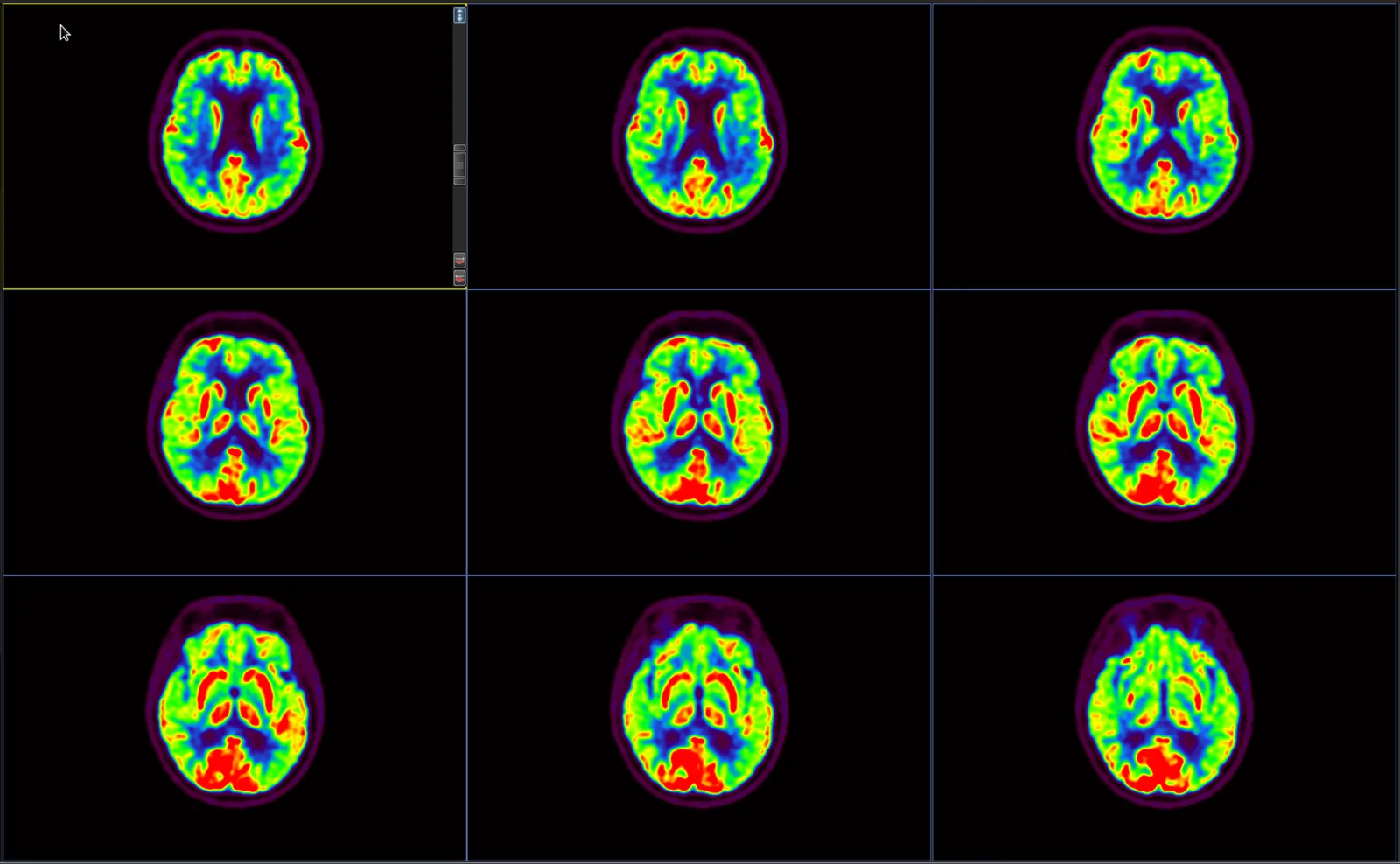
Brain ^18^F-FDG PET imaging. Brain positron emission tomography (PET) scan showing a pattern of diffuse cortical hypometabolism with selective metabolic sparing of the basal ganglia, compatible with metabolic/iatrogenic encephalopathy.

Between the third and fourth weeks, progressive motor recovery was observed, despite the persistence of ideational slowing, impaired attentional functions, and mnemonic deficits. Brain MRI documented multiple areas of T2/FLAIR hyperintensity in the subcortical, deep, and periventricular white matter, partially confluent, consistent with non-specific gliosis (**Figure 1**). An initial neuropsychological assessment using the *Montreal Cognitive Assessment (MoCA)* yielded an overall score below the norm for age and education (1); however, performance validity was questionable due to the patient’s reduced engagement. A brief trial with methylphenidate 10 mg (a two-day test administration) was attempted but produced no clinical improvement and was subsequently discontinued. Given the presence of hypotension, which potentially compromised cerebral perfusion, antihypertensive treatment was tapered and suspended. This decision aimed to optimize cerebral perfusion pressure and address a possible dysautonomic contribution. Concurrently, the diagnostic workup was completed. Lumbar puncture was negative for inflammatory or degenerative processes (2). Regarding molecular neuroimaging, FDG-PET revealed diffuse cortical hypometabolism with sparing of the basal ganglia (**Figure 2**); this finding was further confirmed by a voxel-based semi-quantitative analysis using an age-matched healthy control database. This metabolic profile was compatible with a metabolic/iatrogenic encephalopathy and reversible functional dysfunction. Notably, the absence of focal hypermetabolic areas, particularly in the mesial temporal lobes, allowed to exclude active neuroinflammatory or paraneoplastic processes. EEG showed diffuse slow-wave abnormalities with preserved background rhythm.

### Functional recovery and follow-up

Throughout the hospitalization, the patient underwent physical therapy with progressive motor recovery. At discharge, with a markedly improved cognitive and psychomotor profile, psychopharmacological therapy consisted solely of pramipexole 0.18 mg TID and quetiapine 25 mg *pro re nata* (PRN). Concurrent medications included: prednisone 5 mg/day, folic acid 5 mg/day, omega-3–1 g/day, pantoprazole 20 mg/day, and analgesic therapy with oxycodone/paracetamol 325 + 5 mg/day (**Table 1**).

**Table 1:** Timeline of the clinical course, oncological status, neuropsychiatric symptoms, and therapeutic interventions.

Period	Oncological status & treatments	Neuropsychiatric symptoms	Diagnostic workup & interventions
From 2021	Diagnosis of endometrial carcinoma; surgery and adjuvant chemotherapy (May 2021).	Anxious-depressive disorder with fluctuating affective symptoms, residual anxiety, and emotional vulnerability.	Incomplete clinical response to various psychopharmacological treatments, including antipsychotics (olanzapine, brexpiprazole), antidepressants (citalopram, vortioxetine, escitalopram), and anxiolytics (benzodiazepines and pregabalin).
April 2023 – June 2024	Peritoneal progression (April 2023); initiates Pembrolizumab + Lenvatinib (favorable response). Debulking surgery + HIPEC (Sept 2023); combined oncology therapy resumed (Oct 2023).	Symptoms closely associated with oncological status. Progressive worsening with akathisia and a sub-confusional state after the introduction of biological therapy.	Managed with home regimen: Lenvatinib (10 mg/day), Pembrolizumab (200 mg every 21 days), Escitalopram (15 mg/day), Delorazepam (4 mg/day), Pregabalin (75 mg TID), Olmesartan (40 mg/day), Prednisone (5 mg/day), Oxycodone/Paracetamol (325 + 5 mg/day).
Week 1 (Admission)	Multidisciplinary oncology evaluation: Lenvatinib suspended, next Pembrolizumab dose postponed.	Severe psychomotor retardation, disorientation in time and space, fluctuating sub-confusional state, slurred/hypophonic speech, generalized fine tremor, severe akathisia, and clinophilia.	Admission to SPDC (July 2024). Urgent brain CT (negative). Escitalopram discontinued. IV Flumazenil administered in fractionated doses (positive response in consciousness/orientation). Controlled benzodiazepine/pregabalin décalage with long-half-life diazepam. Folic acid and omega-3 started.
Week 2	Oncological treatment held.	Improvement in consciousness and responsiveness; persistence of disabling akathisia, tremors, and extrapyramidal features.	Completion of benzodiazepine withdrawal. Dopaminergic support with Pramipexole initiated (0.18 mg/day titrated to 0.18 mg TID), resulting in progressive reactivation.
Weeks 3–4	Oncological treatment held.	Progressive motor recovery; persistence of ideational slowing, impaired attention, and mnemonic deficits. Persistent hypotension.	Diagnostic workup completion: Brain MRI (non-specific gliosis); MoCA (low score, questionable validity); Brain FDG-PET (diffuse cortical hypometabolism with basal ganglia sparing); EEG (diffuse slow-wave abnormalities); Lumbar puncture (negative). Antihypertensive (olmesartan) tapered and suspended. Methylphenidate brief trial (2-day test administration, ineffective, discontinued). Physical therapy ongoing.
Discharge	Permanent discontinuation of Pembrolizumab + Lenvatinib decided.	Markedly improved cognitive and psychomotor profile.	Discharge from SPDC. Psychopharmacological therapy at discharge consists solely of Pramipexole 0.18 mg TID and Quetiapine 25 mg PRN (plus somatic medications: prednisone 5 mg/day, folic acid 5 mg/day, omega-3–1 g/day, pantoprazole 20 mg/day, oxycodone/paracetamol 325 + 5 mg/day).
Post-Discharge Rehabilitation	Oncology/Psychiatry follow-up.	Independent ambulation, neurocognitive training, affective-anxiety improvement.	Transfer to intensive rehabilitation. Low-dose Quetiapine (75 mg/day) briefly reintroduced. Neuropsychological reassessment shows significant MoCA improvement, with residual executive susceptibility to cognitive overload.

The patient was subsequently transferred to intensive rehabilitation, achieving independent ambulation, undergoing structured neurocognitive training, and showing affective-anxiety improvement. Low-dose quetiapine (75 mg/day) was briefly reintroduced. Neuropsychological reassessment demonstrated significant improvement in MoCA scores, though a heightened susceptibility to cognitive overload in executive control processes persisted. This was evidenced by sub-normal performance in attentional switching tasks (Trail Making Test – Parts B and B-A; 3) and mildly slowed response times during interference inhibition (Stroop Test; 4). Deficits were also noted in psychomotor speed (Trail Making Test – Part A; 3). The remaining cognitive domains assessed were within normal limits (selective attention, auditory-verbal and visuospatial short-term memory, auditory-verbal working memory, cognitive flexibility, anterograde verbal long-term memory, lexical retrieval, visuospatial and visuoconstructive abilities, and facial emotion recognition). Subjectively, since returning home, the patient reported good cognitive efficiency in activities of daily living (ADLs).

## Scientific literature review

The increasing use of ICIs and MKIs has significantly improved the prognosis of various solid tumors; however, it has also introduced a novel spectrum of neurological and psychiatric adverse events. These complications may be mediated by aberrant immune mechanisms, endothelial dysfunction, or interactions with pre-existing autoimmune comorbidities. Among the most widely employed therapeutic combinations, pembrolizumab (an anti-PD-1 agent) and lenvatinib (a VEGFR/FGFR/RET inhibitor) have garnered increasing clinical attention due to the potential risk of encephalopathies and neuropsychiatric disorders.

### Pembrolizumab: immune-mediated neuro-psychiatric manifestations

ICIs, including pembrolizumab, can induce neurological *immune-related adverse events (irAEs)* ranging from encephalitis and myelitis to peripheral neuropathies and altered mental status. Although rare, these events can be clinically significant, necessitating treatment discontinuation and immunosuppressive therapy.

Sono et al. (5) described a case of neuromyelitis optica spectrum disorder with anti-AQP4 antibody positivity, which emerged during pembrolizumab treatment for lung carcinoma and was reversible with corticosteroids and drug withdrawal. The analysis suggested a mechanism of immune cross-reactivity between tumor antigens and the central nervous system.

Modi et al. (6) reported a case of autoimmune encephalitis associated with pembrolizumab, characterized by acute alteration of mental status and cerebrospinal fluid pleocytosis in the absence of brain neoplasm or infection, with rapid resolution following steroid therapy.

Jono et al. (7) described neuropsychiatric disorders (tremor, confusion, anxiety, and depression) in a patient with pre-existing lupus, with complete resolution after ICI discontinuation; this case highlights how pre-existing autoimmune conditions may increase vulnerability to neuropsychiatric symptoms induced by PD-1 inhibitors.

Similarly, Youjiang Tan et al. (8) described a case of pembrolizumab-induced encephalitis, with a particular focus on the differential diagnosis against Wernicke’s encephalopathy.

A further case of autoimmune encephalitis during pembrolizumab therapy was described by Civardi et al. (9).

Overall, the literature suggests that pembrolizumab can precipitate complex neuro-immune syndromes, which are often reversible with early recognition and immunosuppressive management.

### Lenvatinib: vascular encephalopathy and posterior reversible encephalopathy syndrome

Lenvatinib is generally well-tolerated; however, cases of reversible encephalopathy compatible with PRES have been reported. These are likely linked to endothelial dysfunction and blood pressure instability induced by VEGF inhibition.

Mahajan et al. (10) described a case of atypical PRES in a woman with metastatic thyroid carcinoma treated with lenvatinib, characterized by confusion, seizures, and MRI lesions consistent with vasogenic edema, which were fully reversible following drug discontinuation and blood pressure management.

Similarly, Osawa et al. (11) reported PRES in a patient receiving lenvatinib for extensive loco-regional metastases. The condition resolved upon treatment suspension, suggesting that endothelial impairment plays a central role in the pathophysiological mechanism.

### Clinical synthesis

Overall, the available evidence indicates that:

- ICIs can induce autoimmune encephalopathies and neuropsychiatric disorders.
- Lenvatinib has been associated with PRES and vasogenic cerebral dysfunction.
- Both agents, individually or in combination, can contribute to clinical presentations of cognitive impairment, psychomotor slowing, and mood alterations.
- Treatment discontinuation and immunomodulatory/supportive management often lead to significant clinical improvement.

These data support the interpretation of our case as a potential multifactorial neuro-iatrogenic manifestation, in which oncological polypharmacy, individual susceptibility, and immune-inflammatory alterations interacted to determine a reversible encephalopathic state.

## Discussion

The presented clinical report highlights the diagnostic and management complexity of neuropsychiatric syndromes in oncological patients undergoing immuno-targeted therapies, particularly when psychiatric vulnerability and polypharmacy coexist. The patient developed a subacute organic mental disorder characterized by confusion, ideomotor slowing, akathisia, cognitive deficits, and marked clinophilia, occurring in temporal relationship with the initiation of the pembrolizumab-lenvatinib combination. This correlation, coupled with the progressive clinical improvement following the discontinuation of oncological agents and psychopharmacological washout, strongly supports the hypothesis of a reversible iatrogenic toxicity.

During hospitalization, the absence of significant findings on structural neuroimaging and cerebrospinal fluid analysis allowed for the exclusion of differential diagnoses, such as primary neuroinflammatory or neurodegenerative processes. Concurrently, the transient response to flumazenil suggested a contribution of GABAergic redundancy resulting from prolonged benzodiazepine use, integrating into a multifactorial mechanism. The failure of methylphenidate to improve the cognitive profile further pointed toward an organic encephalopathic etiology rather than a primary attentional disorder or a fronto-striato-thalamic hypoactivation reversible by dopaminergic agents. In this context, akathisia emerged as a significant manifestation: the clinical response to pramipexole – a dopaminergic agent known for its efficacy in iatrogenic and withdrawal-related forms – provides further clinical evidence in defining the nature of the syndrome.

The pathogenetic mechanism appears plausibly synergistic: on one hand, pembrolizumab may induce immune dysregulation and autoimmune phenomena affecting the central nervous system; on the other, lenvatinib is associated with endothelial dysfunction and potential patterns of reversible encephalopathy (12). In a patient undergoing concurrent treatment with psychotropic drugs, these effects may be amplified, manifesting as subtle and fluctuating clinical features. This scenario illustrates how the overlap of immunological, vascular, and neuropharmacological effects can generate complex encephalopathiform syndromes, with prominent motor and executive manifestations that risk being misinterpreted as a primary psychiatric exacerbation.

The favorable clinical evolution – supported by the discontinuation of oncological therapies, gradual benzodiazepine detoxification, the targeted introduction of symptomatic treatments (flumazenil, pramipexole), and a neurocognitive and motor rehabilitation program – reinforces the reversible nature of the syndrome. Of particular clinical significance is the recovery documented during neuropsychological reassessment, which serves as an indicator not only of functional reversibility but also of the prognostic value of systematic cognitive monitoring.

### Clinical implications

This case underscores the importance of systematic neuropsychiatric monitoring in patients undergoing treatment with immune checkpoint inhibitors and multikinase inhibitors, particularly when pre-existing psychiatric vulnerabilities and polypharmacy are present. Neuropsychiatric symptoms in this population may be subtle, fluctuating, and easily misinterpreted as primary psychiatric disorders, potentially delaying appropriate intervention.

The findings highlight the clinical utility of a multidisciplinary approach integrating psychiatry, oncology, neurology, neuropsychology and nuclear medicine to ensure accurate diagnosis and timely management. In particular, structured pharmacological review and washout strategies may play a crucial role in disentangling complex iatrogenic conditions.

Moreover, this case brings to light a profound clinical and ethical dilemma often encountered in contemporary neuro-oncology: balancing aggressive, life-prolonging anti-cancer treatments with the preservation of a patient’s neuropsychiatric well-being and overall quality of life. In our patient, the pembrolizumab-lenvatinib combination had achieved remarkable oncological success, including tumor debulking and an optimal response of the peritoneal recurrence. However, the emergence of severe iatrogenic encephalopathy and disabling akathisia forced a critical re-evaluation of the risk-benefit ratio. Clinicians are frequently caught between the mandate to maintain active oncology regimens and the imperative to stop treatment when central nervous system toxicities severely impair autonomy, cognitive baseline, and emotional stability. In our clinical experience, managing this delicate trade-off was successfully achieved only through an intensive, constant, and case-specific interaction with the oncology team. Rather than relying on isolated cross-consultations, joint re-evaluations allowed us to balance real-time oncological priorities with psychiatric safety. Deciding on the permanent discontinuation of an effective oncological therapy requires a meticulous, shared decision-making process where neuropsychiatric safety and somatic survival are weighed equally, recognizing that a favorable oncological response cannot be sustained at the cost of a catastrophic decline in psychiatric and functional well-being.

Furthermore, the use of targeted psychopharmacological interventions—such as flumazenil for suspected GABAergic overactivity and dopaminergic agents for extrapyramidal symptoms—may provide both diagnostic and therapeutic benefits. Early recognition and intervention can improve functional outcomes, preserve treatment adherence, and reduce morbidity in complex psycho-oncological patients.

### Study limitations

The interpretation of the clinical picture is necessarily limited by the coexistence of multiple therapeutic interventions and their temporal overlap during hospitalization, which constrains the attribution of the observed improvement to any single component.

Although the diagnostic workup excluded major structural and inflammatory conditions, the absence of disease-specific biomarkers—particularly for immune-mediated neurotoxicity—limits the possibility of a more precise pathophysiological characterization. Neuropsychological assessment was further limited by the patient’s fluctuating level of participation and reduced tolerance to cognitive effort during the acute phase, with potential effects on performance validity. This introduces a degree of uncertainty in the interpretation of both baseline deficits and subsequent improvements, as changes over time may partially reflect differences in attentional engagement and psychomotor activation rather than exclusively underlying cognitive recovery.

## Conclusions

The case we described highlights the clinical significance of vigilant neuropsychiatric monitoring in oncological patients undergoing innovative treatments such as pembrolizumab and lenvatinib, particularly in the presence of pre-existing psychiatric conditions and polypharmacy.

The emergence of subacute neuropsychiatric symptoms – often subtle or atypical – necessitates constant, multidisciplinary monitoring to ensure the timely recognition of toxicity signals and the rapid implementation of appropriate diagnostic and therapeutic strategies.

In this patient, the onset of a subacute organic mental disorder – characterized by cognitive impairment, psychomotor slowing, and akathisia – occurred in close temporal relationship with the initiation of immuno-targeted therapy and resolved progressively following the discontinuation of oncological agents and the optimization of psychopharmacological management. It could be noteworthy that the adverse event regressed through psychiatric pharmacological support and oncological treatment suspension, without necessitating the high-dose immunosuppressive therapies (e.g., systemic corticosteroids) typically recommended for immune-related toxicities. This clinical course, coupled with the unremarkable diagnostic findings and the subsequent normalization of the neurocognitive profile, suggests a reversible iatrogenic syndrome. This is consistent with existing literature regarding the neurological and psychiatric toxicities associated with ICIs and MKIs.

Timely management, which included pharmacological washout, the targeted use of symptomatic medications, and an integrated rehabilitation program, enabled a complete functional recovery.

Beyond its clinical value, this case emphasizes interdisciplinarity as an essential operational paradigm in contemporary medicine. Collaboration among oncologists, psychiatrists, neurologists, and neuropsychologists facilitates not only the superior management of complex adverse events but also a deeper understanding of the biological mechanisms underlying neuropsychiatric toxicity.

Indeed, the early identification of neuropsychiatric adverse events allows for corrective measures, such as the temporary suspension of agents or the modulation of psychopharmacological therapy, which in most cases promotes the reversibility of the clinical presentation.

Looking forward, the systematic collection of similar cases and the development of dedicated neuropsychiatric monitoring protocols will contribute to the definition of increasingly personalized and safe therapeutic strategies.

In conclusion, neuropsychiatric surveillance is not merely an ancillary component but an indispensable pillar in the management of complex oncological patients treated with drugs that have a high impact on the central nervous system.

## Data Availability

The original contributions presented in the study are included in the article/supplementary material, further inquiries can be directed to the corresponding author/s.
